# Th17 in Animal Models of Rheumatoid Arthritis

**DOI:** 10.3390/jcm6070073

**Published:** 2017-07-21

**Authors:** Motomu Hashimoto

**Affiliations:** Department of Advanced Medicine for Rheumatic Diseases, Graduate School of Medicine, Kyoto University, Sakyoku Syogoin Kawaramachi 54, Kyoto 606-8507, Japan; mohashim@kuhp.kyoto-u.ac.jp; Tel.: +81-75-751-3877; Fax: +81-75-751-3885

**Keywords:** IL-17-secreting helper CD4 T cells (Th17 cells), animal models, rheumatoid arthritis, synovial fibroblasts, regulatory T cells

## Abstract

IL-17-secreting helper CD4 T cells (Th17 cells) constitute a newly identified subset of helper CD4 T cells that play a key role in the development of rheumatoid arthritis (RA) in its animal models. Recently, several models of spontaneous RA, which elucidate the mechanism of RA onset, have been discovered. These animal models shed new light on the role of Th17 in the development of autoimmune arthritis. Th17 cells coordinate inflammation and promote joint destruction, acting on various cells, including neutrophils, macrophages, synovial fibroblasts, and osteoclasts. Regulatory T cells cannot control Th17 cells under conditions of inflammation. In this review, the pathogenic role of Th17 cells in arthritis development, which was revealed by the recent animal models of RA, is discussed.

## 1. A Brief History of the RA Theory

**Figure 1:**
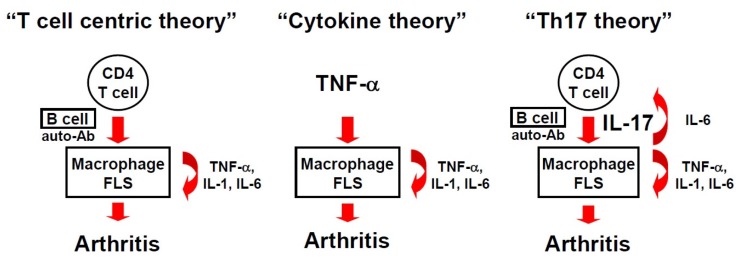
Historical transition of the theories for rheumatoid arthritis (RA) development. AutoAbs, autoantibodies; fibroblast-like synoviocytes (FLSs), fibroblast-like synoviocytes.

Rheumatoid arthritis (RA) is an autoimmune disease, which is characterized by the infiltration of T cells, B cells, macrophages, and synovial fibroblasts into the synovial membrane, leading to joint destruction [1]. Although the etiology of RA remains elusive, it has been classically theorized that CD4 T cells are critical players in the pathogenesis of RA [2]. This notion was supported by several observations: (i) the main cell types that infiltrate the synovial membrane in RA synovitis are CD4 T cells and macrophages [3]; (ii) genes in the human leukocyte antigen (HLA) region remain the most powerful disease risk genes in RA [4]; and (iii) the sera of patients with RA contain abundant autoantibodies [1]. These results suggest that CD4 T cell-mediated autoimmunity is central to the pathogenesis of RA (Figure 1) [2]. 

However, this “T cell-centric theory” of RA pathogenesis has been challenged in the past few decades because of several findings: (i) the concentration of T cell-derived cytokines (IFN-γ, IL-2, and so on) is low in RA synovium compared with those of the abundant macrophage- and fibroblast-derived cytokines (TNF-α, IL-1, IL-6, and so on) [5]; (ii) CD4 T-cell depletion therapy failed to improve RA outcome in clinical trials [6]; and (iii) the deficiency of Th1-promoting cytokines (IL-12, IFN-γ, and so on) paradoxically exacerbated arthritis in animal models of RA (the “Th1 paradox”) [7]. 

After obtaining these findings, the “T cell-centric theory” was questioned, and a new “cytokine theory” of RA pathogenesis emerged [8] (Figure 1). In the new proposition, the key mediators of RA are pro-inflammatory cytokines, which are derived from macrophages and fibroblast-like synoviocytes (FLSs), such as TNF-α, IL-1, and IL-6 [9,10]. Macrophages and FLSs are abundant sources of these pro-inflammatory cytokines, which serve as autologous stimulators of other cytokines, and thereby prolong synovitis in a T cell-independent manner. The development of arthritis in mice that were genetically engineered to overexpress TNF-α, IL-1, or IL-6 supported this hypothesis [11,12,13]. The new proposition resulted in the success of “anti-cytokine therapy”, such as the anti-TNF-α therapy or the anti-IL-6 therapy for RA, which has revolutionized current RA treatment [9]. However, the fact that RA cannot be “cured” even after anti-cytokine therapies suggests that a more integrated view of RA pathogenesis is required.

In 2005, the discovery of Th17 cells provided new insights into how T cells participate in the initiation and prolongation of RA, and led to the revival of the “T cell-centric theory” of RA pathogenesis, as well as the proposal of the new “Th17 theory” [14,15] (Figure 1). After the discovery of CD4 T cells that produced IL-17 in the RA synovium [16,17], studies using animal models revealed that Th17 cells are a lineage of CD4 T cells that are distinct from classical Th1 or Th2 cells, and play key roles in various autoimmune and inflammatory diseases [14,15]. The Th17 cells that express the master transcription factor RORγt are induced by TGF-β and IL-6 to differentiate in vitro, and are expanded by IL-23, IL-1, and TNF-α [18]. Th17 differentiation is cross-regulated by Th1 and Th2 cytokines, such as IFN-γ or IL-4 [14,15]. Therefore, the deficiency of Th-1-promoting cytokines, such as IL-12 or IFN-γ, led to the excessive differentiation of Th17 cells, and paradoxically exacerbated RA in the animal models [19]. Thus, the discovery of Th17 cells solved the “Th1 paradox,” and deepened our understanding of the pathogenesis of autoimmune arthritis. 

In this review, the pathogenic role of Th17 cells, which was revealed by the recent animal models of RA, is discussed. 

## 2. Animal Models of RA

### 2.1. Type II Collagen-Induced Arthritis

The injection of cartilage constituents, such as type II collagen in complete Freund’s adjuvant, into genetically susceptible strains of mice induced synovitis and erosion that histologically resembled RA (CIA, collagen-induced arthritis) [20]. The sera of such mice contained abundant autoantibodies against type II collagen, and the disease could be induced in other mice by injecting anti-type II collagen antibodies (CAIA, anti-type II collagen antibody-induced arthritis) [21] (Figure 1). The final effectors of the disease in this animal model were autoantibodies (anti-type II collagen antibody), although CD4 T cells were required for helping in the production of anti-type II collagen antibodies in B cells in the immunized mice (CIA). 

Using mice that were deficient in IL-12 p35, IL-12/IL-23 p40, and IL-23 p19, it was shown that IL-23-driven CD4 T cells, but not IL-12-driven Th1 cells, are the key mediators of CIA, because CIA was suppressed by the deficiency of IL-23 p19 or IL-12/IL-23 p40, and exacerbated by the deficiency of IL-12 p35 [19] (Table 1). The IL-23-driven CD4 T cells secreted IL-17, and were identified as a T cell lineage (Th17) that was distinct from classical Th1 and Th2 cells [14,15,18]. IL-1 and TNF-α were required for arthritis induced by the transfer of anti-type II collagen antibodies (CAIA), while IL-6 was imperative for arthritis induced by the immunization with type II collagen (CIA), because IL-6 was essential for the differentiation of Th17 cells [22,23,24] (Table 1). 

Th17 cells contribute not only to the promotion of joint inflammation, but also to the induction of osteoclast differentiation and bone destruction. It was classically known that Th1- and Th2-derived cytokines, such as IFN-γ and IL-4, have an inhibitory effect on osteoclast differentiation [41]. Using the CIA model, it was shown that IL-17 acts on synoviocytes to induce receptor activator of nuclear factor-kB ligand (RANKL) expression to promote osteoclast differentiation in synergy with other inflammatory cytokines such as TNF-α [41]. Thus, Th17 cells function as potent “osteoclastogenic” helper CD4 T cells that strongly stimulate the differentiation and activation of osteoclasts [42]. 

### 2.2. SKG Mice

SKG mice on a BALB/c background spontaneously developed autoimmune arthritis that resembled human RA in a conventional environment [43]. SKG mice developed not only arthritis, but also extra-articular lesions, such as interstitial pneumonitis as observed in human RA [44]. The primary cause of arthritis in SKG mice is a point mutation in the *ZAP70* gene, which encodes a key signaling molecule in T cells. Abnormal ZAP-70 in SKG mice attenuates T cell receptor (TCR) signaling, and alters the threshold for positive and negative selection in the thymus, thus allowing for the escape of self-reactive CD4 T cells, including arthritogenic CD4 T cells, into the periphery [43]. SKG arthritis is dependent on CD4 T cells, because arthritis can be induced in T cell deficient mice by the adoptive transfer of CD4 T cells [43]. Similar to CIA, SKG arthritis is dependent on pro-inflammatory cytokines, particularly IL-6 [25] (Table 1).

SKG arthritis is dependent on Th17, because SKG CD4 T cells that were deficient in IL-17 failed to induce arthritis upon adoptive transfer into RAG2-deficient mice, while the induction of arthritis was accelerated by the transfer of IFN-γ-deficient CD4 T cells [26] (Table 1). Interestingly, SKG mice spontaneously developed arthritis in a microbially conventional environment but not under specific pathogen-free (SPF) conditions, which suggests the role of environmental factors [45]. The activation of innate immunity via toll like receptors (TLR) (zymosan, polyI:C, mannan), Dectin-1 (zymosan, β-glucan), or the complement system (zymosan, β-glucan, mannan) triggers arthritis even under SPF conditions [45,46]. Dectin-1 signaling on dendritic cells potently induce the production of IL-23 that promotes Th17 differentiation [47], and the dectin-1 agonist β-glucan triggers the development of not only arthritis, but also psoriatic skin lesions, uveitis, or enthesitis, which are similar to human spondyloarthropathies [48]. Arthritis, enthesitis, and ileitis, which were induced by β-glucan, were inhibited by IL-17 deficiency or anti-IL-23 treatment in SKG mice [27]. Complement activation leads to the production of the anaphylatoxin C5a, which enhances the production of IL-6 from macrophages in synergy with the production of other cell surface receptors to further expand Th17 cells [46]. Th17 cells express the chemokine receptor CCR6, and are recruited to the site of inflammation through a CCL20 gradient [49]. 

One of the target antigens, which was recognized by self-reactive SKG CD4 T cells, was identified to be the 60S ribosomal protein L23a (RPL23A) [50]. Upon recognition of the RPL23-A peptide, the SKG CD4 T cells but not the control BALB/c CD4 T cells proliferated, and secreted IL-17. Although the anti-RPL23-A antibody was detected in the sera of SKG mice and in some patients with RA, the autoantibody itself did not have the capacity to induce arthritis in mice. Instead, the adoptive transfer of CD4 T cells that were reactive to RPL23A could induce arthritis, which suggests the direct arthritogenic effect of CD4 T cells [50].

### 2.3. K/BxN Mice

The F1 offspring resulting from the cross between non-obese diabetic (NOD) mice and KRN TCR transgenic mice developed spontaneous arthritis (K/BxN mice) [51,52]. The sera of the mice contained high titers of antibodies against glucose-6 phosphate isomerase (GPI) peptide, and the disease could be induced in other mice by injecting anti-GPI antibody (K/BxN serum-transfer arthritis) [53]. The development of arthritis in K/BxN mice critically depended on the complement system (particularly, C5a), the Fc-γ receptor, inflammatory cytokines such as IL-1 and TNF-α, neutrophils, macrophages, and mast cells [28,54,55,56]. 

Although CD4 T cells were dispensable to arthritis induced by the injection of anti-GPI antibody (K/BxN serum transfer arthritis), autoreactive KRN CD4 T cells were required for the initiation of arthritis in K/BxN mice (Table 1). CD4 T cells that infiltrated the joints in K/BxN mice secreted IL-17, and the deficiency of IL-17 or IL-23 considerably suppressed the development of K/BxN arthritis but not K/BxN serum-transfer arthritis [29,30] (Table 1). The IL-23-Th17 axis regulated the glycosylation profile of autoantibodies, and were responsible for their inflammatory activity [30]. However, there is still a debate on whether Th17 cells are required for the development of K/BxN arthritis, because KRN CD4 T cells that were deficient in Rorc and unable to differentiate into Th17 cells were able to induce arthritis, while KRN CD4 T cells that were deficient in Bcl-6 and unable to differentiate into follicular helper CD4 T cells failed to induce arthritis [57,58]. It is reported that the production of IL-17 from neutrophils also contributed to the induction of K/BxN arthritis in a CD4 T cell-independent manner [59]. 

Studies using K/BxN mice revealed the importance of intestinal microbiota in the development of Th17 cells. Under a germ-free (GF) environment, K/BxN mice lacked lamina propria Th17, and failed to develop arthritis. However, the introduction of a single bacterial species, namely segmented filamentous bacteria (SFB), into the GF animals restored the lamina propria Th17 and the development of arthritis [31,60]. These results suggest that intestinal microbiota is a key regulator of the development of Th17 cells. The regulation of Th17 cells differentiation by intestinal microbiota will be discussed in another article [61] of this review series. 

### 2.4. IL-1 Receptor-Antagonist Knockout Mice

IL-1 receptor antagonist (IL-1Ra) is an endogenous inhibitor of IL-1, and negatively regulates IL-1 activity [62]. IL-1Ra-deficient mice on a BALB/c background spontaneously developed chronic inflammatory polyarthritis [12]. Histologically, they exhibited marked synovial and periarticular inflammation with bone erosion. Serologically, they developed the rheumatoid factor, anti-type II collagen antibody, and anti-dsDNA antibody. 

Although the overexpression of IL-1 could lead to the activation and proliferation of synoviocytes in an autocrine or paracrine manner [9,62], arthritis in this animal model was dependent on CD4 T cells [12]. The expression of IL-17 or IL-23 was greatly enhanced in IL-1Ra KO mice, and arthritis was inhibited during IL-17 deficiency, or IL-23 blockade by anti-p19 inhibited arthritis development [32,33,34] (Table 1). IL-1 preferentially expanded Th17 cells because Th17 cells express high levels of IL-1 receptor 1 [63]. IL-1 also expanded Th17 cells by enhancing the cognate interaction between CD4 T cells and antigen presenting cells (APCs) through the upregulation of costimulatory molecules [33]. 

### 2.5. gp130 ^F759/F759^ Knock-in Mice

The receptor subunit glycoprotein 130 (gp130) mediates signal transduction by IL-6 family cytokines such as IL-6, IL-11, LIF, IL-27, and IL-35, through signal transducer and activator of transcription 3 (STAT3) STAT3 and/or Src homology region 2 domain-containing phosphatase 2 (SHP2) signal transductions [64]. In gp130 ^F759/F759^ knock-in (gp130 F759) mice, tyrosine 759 (Y759 in human gp130) in the SHP2-binding site of gp130 is replaced with phenylalanine (F) [13]. Because tyrosine 759 is involved in the extracellular-signal-regulated kinase (ERK) signaling pathway mediated by gp130, and is essential for the suppressor of cytokine signaling (SOCS)-mediated negative feedback loop of STAT3 activation, STAT3 signaling is prolonged in gp130 F759 mice [64]. gp130 F759 mice suffer from autoimmune arthritis that clinically resembles RA [13].

In gp130 F759 mice, the activation-induced cell death (AICD) of CD4 T cells was attenuated because prolonged IL-6/gp130 signaling downregulated Fas and Fas ligand expression. As a result, gp130 F759 mice had expanded effector memory CD4 T cells [65]. Th17 cells were expanded in gp130 F759 mice, because IL-6/gp130 signaling promoted Th17 differentiation [35]. Arthritis in gp130 F759 mice was considerably inhibited by the deficiency of IL-17 or IL-6, and the combination of IL-17 and IL-6 markedly enhanced the production of IL-6 from FLS [36]. Thus, a positive feedback loop between IL-6 and IL-17, which was triggered by Th17 cells, was responsible for the development of arthritis in gp130 F759 mice [36] (Figure 1). 

### 2.6. TNF-α Transgenic Mice

The systemic overexpression of TNF-α causes arthritis with subchondral erosions (in TNF-α transgenic mice) [11,66]. In contrast to the abovementioned animal models, CD4 T cells were not required for the development of arthritis in this animal model. Instead, arthritis could be induced in other mice by the adoptive transfer of fibroblast-like synoviocytes from TNF-α transgenic mice [67]. The synoviocytes that overexpressed TNF-α acquired an aggressive phenotype, and exhibited increased proliferation and decreased adhesion to the extracellular matrix for mediating immunity-independent arthritis, which is driven by TNF-α [67] (Figure 1). In TNF-α transgenic mice, arthritis was completely inhibited by IL-1 deficiency, suggesting that IL-1 acts downstream of TNF-α, while IL-6 was not required for the development of arthritis in this animal model [23,37] (Table 1). 

TNF-α strongly promotes osteoclast differentiation in vivo and in vitro synergistically with RANKL (receptor activator of NF-κB ligand), and causes inflammatory bone resorption [68]. Although anti-IL-17 therapy had only minor effects on joint inflammation induced by TNF-α, it effectively reduced bone erosion in TNF-a transgenic mice [69]. This result suggests that although arthritis in TNF-α transgenic mice can develop in a T cell-independent manner, IL-17 may also contribute to bone destruction, which is mediated by TNF-α. 

## 3. Role of IL-17 in the Development of Arthritis

Studies of these animal models indicate that Th17 cells could be major common factors responsible for the development of autoimmune arthritis in animal models. The potent arthritogenic effect of Th17 cells mainly lies in the pleiotropic effect of IL-17A (IL-17), which is produced by Th17 cells and acts on a variety of cells that constitute the synovial tissue [70,71]: (i) IL-17 acts on macrophages and synovial fibroblasts, and synergistically enhances the production of inflammatory cytokines, such as TNF-α, IL-1, and IL-6 [36,72]; (ii) IL-17 recruits neutrophils to the site of inflammation, and enhances granulopoiesis in order to cause neutrophil-mediated inflammation [71]; and (iii) IL-17 promotes osteoclast differentiation, which leads to bone erosion and cartilage destruction [41]. In addition, IL-22 and IL-21, which are produced by Th17 cells, alter the glycosylation of autoantibodies and provide them with inflammatory properties [30]. Thus, Th17 cells are potent mediators of arthritis, which coordinate tissue inflammation, cartilage damage, and bone erosion. Although Th17 cells are involved in the development of arthritis in many animal models, it should be noted that Th17 cells may not be a prerequisite for all models of arthritis, because RA is a heterogeneous disease which is mediated by a variety of factors, including the dysregulated activation of T cells and B cells or the dysregulated production of inflammatory cytokines from synoviocytes [73]. 

## 4. Interplay between Th17 Cells and Regulatory T Cells

The activation of highly inflammatory Th17 cells should be controlled in normal physiological conditions to prevent the development of autoimmune diseases. Regulatory T cells (Tregs) are agents that control the activation of effector CD4 T cells and prevent the development of autoimmune disease [74]. Tregs express the master transcription factor Foxp3, and upregulate the expression of CD25 and CTLA-4 on their surfaces in order to suppress the activation of effector CD4 T cells in a cell-contact-dependent manner [74]. There are two types of Tregs: naturally occurring Tregs (nTregs), which are derived from the thymus, and induced Tregs (iTregs), which are induced to differentiate from naïve T cells in the periphery. It is well established that Tregs efficiently suppress the activation of Th1 or Th2 cells. On the other hand, Th17 cells are less susceptible to Treg-mediated suppression than Th1 or Th2 cells. There may be several reasons for this: (i) iTregs and Th17 cells share some of their developmental pathways, however, iTregs are induced to differentiate by TGF-β alone, while Th17 cells are induced to differentiate by IL-6 and TGF-β [24,75]; (ii) T reg-mediated suppression is attenuated in the presence of Th17-related cytokines, such as IL-6, IL-23, IL-1, and TNF-α [76,77,78,79]; and (iii) Tregs can be converted into Th17 cells in the presence of IL-6 at the site of inflammation [80]. Using the reporter system for fate-mapping of cells expressing Foxp3 in CIA, it was shown that some of the Treg (CD25^low^ Foxp3+) cells lose their Foxp3 expression and undergo transdifferentiation into Th17 cells in tissues with abundant IL-6 [80]. The Th17 cells that are derived from Tregs are more osteoclastogenic than the naïve Th17 cells that are derived from T cells [80]. These findings suggest that the imbalance between Th17 cells and Tregs may contribute to RA pathology, and Foxp3 instability might be involved in the generation of pathogenic Th17 cells in inflammatory tissues [81,82]. 

## 5. Th17 Cells in Mice and Humans

Although Th17 cells play a key role in the development of arthritis in these animal models, the role of Th17 cells in the development of RA in humans is controversial [83]. One reason may be that Th17 cells of mice and humans have different developmental pathways, and IL-17 can be produced from sources other than Th17 cells [84,85,86,87]. Several reports confirm that IL-17 is upregulated in joint tissues in the early phase of RA [88], while other reports suggest that Th1 cells, but not Th17 cells, are the predominant cells in established synovitis related to RA [89]. Clinical trials of therapy with anti-IL-17 antibody indicated partially reduced symptoms of arthritis in RA, while it was highly effective for psoriasis or psoriatic arthritis (PsA) [38,39] (Table 1). Therapy with anti-IL-12/IL-23 p40 antibody was also effective for psoriasis or PsA, but not for RA [40,90] (Table 1). These results suggest that the Th17/IL-23 axis is more relevant to psoriasis or PsA than to human RA. Consistent with human studies, psoriasis-like skin lesions, but not arthritis, were observed in mice that underwent IL-17 gene transfer in vivo [91]. Nevertheless, it should be noticed that the mice into which the *IL-17* gene was transferred showed pathological bone resorption with the absence of clinically noticeable joint inflammation, and developed severe arthritis when the mice were immunized with type II collagen (CIA) [91]. Therefore, Th17 cells or IL-17 are indeed involved in the resistance to treatment and bone destruction in autoimmune arthritis, including RA and PsA [92,93]. The similarities and differences between Th17 cells of humans and mice will be discussed in another article [94] of this review series [95]. 

## 6. Conclusions

Th17 cells play a crucial role in RA development in the animal models of RA. Th17 cells coordinate joint inflammation, mediate bone destruction, and play an important role in the development of autoimmune arthritis. Animal models of RA are instrumental in clarifying the mechanism whereby Th17 cells are induced to differentiate and are activated to mediate autoimmune arthritis. 

The severity of arthritis after the inhibition of the indicated cytokines by genetic modification (knockout) or by treatment with neutralizing anti-cytokine antibodies is shown in Table 1.

## Figures and Tables

**Table 1:** Regulation of the development of arthritis by the inhibition of cytokines in animal models of rheumatoid arthritis (RA) and in human RA and psoriatic arthritis (PsA).

Models	Effectors	IL-17	IFN-γ	TNF-α	IL-1	IL-6	IL-12 p35	IL-12/IL-23 p40	IL-23 p19	Reference
CIA	CD4 T, autoAbs	↓↓↓	↑	↓↓	↓↓↓	↓↓↓	↑	↓↓↓	↓↓↓	[7,19,23]
CAIA	autoAbs	ND	ND	↓↓	↓↓↓	→	ND	ND	ND	[22]
SKG	CD4 T	↓↓↓	↑	↓↓	↓↓	↓↓↓	ND	ND	↓↓↓	[25,26,27]
K/BxN	CD4 T, autoAbs	↓	→	↓↓	↓↓↓	ND	→	ND	↓	[28,29,30,31]
K/BxN serum transfer	autoAbs	→	ND	↓↓	↓↓↓	→	ND	ND	ND	[28,29]
IL-1Ra KO	CD4 T, synoviocytes	↓↓↓	ND	↓↓↓	↓↓↓	ND	ND	ND	↓↓↓	[32,33,34]
Gp130 F759	CD4 T, synoviocytes	↓↓↓	ND	ND	ND	↓↓↓	ND	ND	ND	[35,36]
TNF-α Tg	synoviocytes	(↓)	ND	↓↓↓	↓↓↓	→	ND	ND	ND	[23,37]
RA	CD4 T, autoAbs, synoviocytes	↓	→	↓↓↓	↓	↓↓↓	ND	→	ND	[9,38]
PsA	CD4 T, synoviocytes	↓↓↓	ND	↓↓↓	↓↓	ND	ND	↓↓↓	ND	[39,40]

Note: ↓↓↓, marked suppression of arthritis; ↓↓, moderate suppression of arthritis; ↓, partial suppression of arthritis; (↓), no change in arthritis, but inhibition of bone destruction; ↑, exacerbation of arthritis; →, minimal or no change; RA, rheumatoid arthritis; PsA, psoriatic arthritis; CIA, arthritis induced by immunization with type II collagen; CAIA, arthritis induced by the transfer of anti-type II collagen antibody; K/BxN serum-transfer, arthritis induced by the transfer of anti-GPI antibody; IL-1Ra KO, IL-1 receptor-antagonist knockout mouse; F759 KI, gp130 ^F759/F759^ knock-in mouse; TNF-α Tg, TNF-α transgenic mouse; autoAbs, autoantibodies; ND, not determined.

## References

[1] Firestein G.S. (2003). Evolving concepts of rheumatoid arthritis. Nature.

[2] Lundy S.K., Sarkar S., Tesmer L.A., Fox D.A. (2007). Cells of the synovium in rheumatoid arthritis. T lymphocytes. Arthritis Res. Ther..

[3] Janossy G., Panayi G., Duke O., Bofill M., Poulter L.W., Goldstein G. (1981). Rheumatoid arthritis: A disease of T-lymphocyte/macrophage immunoregulation. Lancet.

[4] Klareskog L., Forsum U., Scheynius A., Kabelitz D., Wigzell H. (1982). Evidence in support of a self-perpetuating HLA-DR-dependent delayed-type cell reaction in rheumatoid arthritis. Proc. Natl. Acad. Sci. USA.

[5] Firestein G.S., Zvaifler N.J. (1990). How important are T cells in chronic rheumatoid synovitis?. Arthritis Rheum..

[6] Wendling D., Racadot E., Wijdenes J., Sibilia J., Flipo R.M., Cantagrel A., Miossec P., Eschard J.P., Macro M., Bertin P. (1998). A randomized, double blind, placebo controlled multicenter trial of murine anti-CD4 monoclonal antibody therapy in rheumatoid arthritis. J. Rheumatol..

[7] Vermeire K., Heremans H., Vandeputte M., Huang S., Billiau A., Matthys P. (1997). Accelerated collagen-induced arthritis in IFN-γ receptor-deficient mice. J. Immunol..

[8] Firestein G.S., Zvaifler N.J. (2002). How important are T cells in chronic rheumatoid synovitis? II. T cell-independent mechanisms from beginning to end. Arthritis Rheum..

[9] Feldmann M., Brennan F.M., Maini R.N. (1996). Role of cytokines in rheumatoid arthritis. Annu. Rev. Immunol..

[10] Udalova I.A., Mantovani A., Feldmann M. (2016). Macrophage heterogeneity in the context of rheumatoid arthritis. Nat. Rev. Rheumatol..

[11] Keffer J., Probert L., Cazlaris H., Georgopoulos S., Kaslaris E., Kioussis D., Kollias G. (1991). Transgenic mice expressing human tumour necrosis factor: A predictive genetic model of arthritis. EMBO J..

[12] Horai R., Saijo S., Tanioka H., Nakae S., Sudo K., Okahara A., Ikuse T., Asano M., Iwakura Y. (2000). Development of chronic inflammatory arthropathy resembling rheumatoid arthritis in interleukin 1 receptor antagonist-deficient mice. J. Exp. Med..

[13] Atsumi T., Ishihara K., Kamimura D., Ikushima H., Ohtani T., Hirota S., Kobayashi H., Park S.J., Saeki Y., Kitamura Y. (2002). A point mutation of Tyr-759 in interleukin 6 family cytokine receptor subunit gp130 causes autoimmune arthritis. J. Exp. Med..

[14] Harrington L.E., Hatton R.D., Mangan P.R., Turner H., Murphy T.L., Murphy K.M., Weaver C.T. (2005). Interleukin 17-producing CD4+ effector T cells develop via a lineage distinct from the T helper type 1 and 2 lineages. Nat. Immunol..

[15] Park H., Li Z., Yang X.O., Chang S.H., Nurieva R., Wang Y.H., Wang Y., Hood L., Zhu Z., Tian Q. (2005). A distinct lineage of CD4 T cells regulates tissue inflammation by producing interleukin 17. Nat. Immunol..

[16] Kotake S., Udagawa N., Takahashi N., Matsuzaki K., Itoh K., Ishiyama S., Saito S., Inoue K., Kamatani N., Gillespie M.T. (1999). IL-17 in synovial fluids from patients with rheumatoid arthritis is a potent stimulator of osteoclastogenesis. J. Clin. Investig..

[17] Chabaud M., Durand J.M., Buchs N., Fossiez F., Page G., Frappart L., Miossec P. (1999). Human interleukin-17: A T cell-derived proinflammatory cytokine produced by the rheumatoid synovium. Arthritis Rheum..

[18] Ivanov I.I., McKenzie B.S., Zhou L., Tadokoro C.E., Lepelley A., Lafaille J.J., Cua D.J., Littman D.R. (2006). The orphan nuclear receptor RORγt directs the differentiation program of proinflammatory IL-17+ T helper cells. Cell.

[19] Murphy C.A., Langrish C.L., Chen Y., Blumenschein W., McClanahan T., Kastelein R.A., Sedgwick J.D., Cua D.J. (2003). Divergent pro- and antiinflammatory roles for IL-23 and IL-12 in joint autoimmune inflammation. J. Exp. Med..

[20] Brand D.D., Latham K.A., Rosloniec E.F. (2007). Collagen-induced arthritis. Nat. Protoc..

[21] Khachigian L.M. (2006). Collagen antibody-induced arthritis. Nat. Protoc..

[22] Kagari T., Doi H., Shimozato T. (2002). The importance of IL-1 β and TNF-α, and the noninvolvement of IL-6, in the development of monoclonal antibody-induced arthritis. J. Immunol..

[23] Alonzi T., Fattori E., Lazzaro D., Costa P., Probert L., Kollias G., De Benedetti F., Poli V., Ciliberto G. (1998). Interleukin 6 is required for the development of collagen-induced arthritis. J. Exp. Med..

[24] Veldhoen M., Hocking R.J., Atkins C.J., Locksley R.M., Stockinger B. (2006). TGFbeta in the context of an inflammatory cytokine milieu supports de novo differentiation of IL-17-producing T cells. Immunity.

[25] Hata H., Sakaguchi N., Yoshitomi H., Iwakura Y., Sekikawa K., Azuma Y., Kanai C., Moriizumi E., Nomura T., Nakamura T. (2004). Distinct contribution of IL-6, TNF-α, IL-1, and IL-10 to T cell–mediated spontaneous autoimmune arthritis in mice. J. Clin. Investig..

[26] Hirota K., Hashimoto M., Yoshitomi H., Tanaka S., Nomura T., Yamaguchi T., Iwakura Y., Sakaguchi N., Sakaguchi S. (2007). T cell self-reactivity forms a cytokine milieu for spontaneous development of IL-17+ Th cells that cause autoimmune arthritis. J. Exp. Med..

[27] Benham H., Rehaume L.M., Hasnain S.Z., Velasco J., Baillet A.C., Ruutu M., Kikly K., Wang R., Tseng H.W., Thomas G.P. (2014). Interleukin-23 mediates the intestinal response to microbial beta-1,3-glucan and the development of spondyloarthritis pathology in SKG mice. Arthritis Rheumatol..

[28] Ji H., Pettit A., Ohmura K., Ortiz-Lopez A., Duchatelle V., Degott C., Gravallese E., Mathis D., Benoist C. (2002). Critical Roles for Interleukin 1 and Tumor Necrosis Factor α in Antibody-induced Arthritis. J. Exp. Med..

[29] Jacobs J.P., Wu H.J., Benoist C., Mathis D. (2009). IL-17-producing T cells can augment autoantibody-induced arthritis. Proc. Natl. Acad. Sci. USA.

[30] Pfeifle R., Rothe T., Ipseiz N., Scherer H.U., Culemann S., Harre U., Ackermann J.A., Seefried M., Kleyer A., Uderhardt S. (2017). Regulation of autoantibody activity by the IL-23-TH17 axis determines the onset of autoimmune disease. Nat. Immunol..

[31] Wu H.J., Ivanov I.I., Darce J., Hattori K., Shima T., Umesaki Y., Littman D.R., Benoist C., Mathis D. (2010). Gut-residing segmented filamentous bacteria drive autoimmune arthritis via T helper 17 cells. Immunity.

[32] Koenders M.I., Devesa I., Marijnissen R.J., Abdollahi-Roodsaz S., Boots A.M., Walgreen B., di Padova F.E., Nicklin M.J., Joosten L.A., van den Berg W.B. (2008). Interleukin-1 drives pathogenic Th17 cells during spontaneous arthritis in interleukin-1 receptor antagonist-deficient mice. Arthritis Rheum..

[33] Nakae S., Saijo S., Horai R., Sudo K., Mori S., Iwakura Y. (2003). IL-17 production from activated T cells is required for the spontaneous development of destructive arthritis in mice deficient in IL-1 receptor antagonist. Proc. Natl. Acad. Sci. USA.

[34] Cho M.L., Kang J.W., Moon Y.M., Nam H.J., Jhun J.Y., Heo S.B., Jin H.T., Min S.Y., Ju J.H., Park K.S. (2006). STAT3 and NF-κB Signal Pathway Is Required for IL-23-Mediated IL-17 Production in Spontaneous Arthritis Animal Model IL-1 Receptor Antagonist-Deficient Mice. J. Immunol..

[35] Nishihara M., Ogura H., Ueda N., Tsuruoka M., Kitabayashi C., Tsuji F., Aono H., Ishihara K., Huseby E., Betz U.A. (2007). IL-6-gp130-STAT3 in T cells directs the development of IL-17+ Th with a minimum effect on that of Treg in the steady state. Int. Immunol..

[36] Ogura H., Murakami M., Okuyama Y., Tsuruoka M., Kitabayashi C., Kanamoto M., Nishihara M., Iwakura Y., Hirano T. (2008). Interleukin-17 promotes autoimmunity by triggering a positive-feedback loop via interleukin-6 induction. Immunity.

[37] Probert L., Plows D., Kontogeorgos G., Kollias G. (1995). The type I interleukin-1 receptor acts in series with tumor necrosis factor (TNF) to induce arthritis in TNF-transgenic mice. Eur. J. Immunol..

[38] Genovese M.C., Durez P., Richards H.B., Supronik J., Dokoupilova E., Mazurov V., Aelion J.A., Lee S.H., Codding C.E., Kellner H. (2013). Efficacy and safety of secukinumab in patients with rheumatoid arthritis: A phase II, dose-finding, double-blind, randomised, placebo controlled study. Ann. Rheum. Dis..

[39] Burkett P.R., Kuchroo V.K. (2016). IL-17 Blockade in Psoriasis. Cell.

[40] Kavanaugh A., Ritchlin C., Rahman P., Puig L., Gottlieb A.B., Li S., Wang Y., Noonan L., Brodmerkel C., Song M. (2014). Ustekinumab, an anti-IL-12/23 p40 monoclonal antibody, inhibits radiographic progression in patients with active psoriatic arthritis: Results of an integrated analysis of radiographic data from the phase 3, multicentre, randomised, double-blind, placebo-controlled PSUMMIT-1 and PSUMMIT-2 trials. Ann. Rheum. Dis..

[41] Sato K., Suematsu A., Okamoto K., Yamaguchi A., Morishita Y., Kadono Y., Tanaka S., Kodama T., Akira S., Iwakura Y. (2006). Th17 functions as an osteoclastogenic helper T cell subset that links T cell activation and bone destruction. J. Exp. Med..

[42] Takayanagi H. (2012). New developments in osteoimmunology. Nat. Rev. Rheumatol..

[43] Sakaguchi N., Takahashi T., Hata H., Nomura T., Tagami T., Yamazaki S., Sakihama T., Matsutani T., Negishi I., Nakatsuru S. (2003). Altered thymic T-cell selection due to a mutation of the ZAP-70 gene causes autoimmune arthritis in mice. Nature.

[44] Keith R.C., Powers J.L., Redente E.F., Sergew A., Martin R.J., Gizinski A., Holers V.M., Sakaguchi S., Riches D.W. (2012). A novel model of rheumatoid arthritis-associated interstitial lung disease in SKG mice. Exp. Lung Res..

[45] Yoshitomi H., Sakaguchi N., Kobayashi K., Brown G.D., Tagami T., Sakihama T., Hirota K., Tanaka S., Nomura T., Miki I. (2005). A role for fungal β-glucans and their receptor Dectin-1 in the induction of autoimmune arthritis in genetically susceptible mice. J. Exp. Med..

[46] Hashimoto M., Hirota K., Yoshitomi H., Maeda S., Teradaira S., Akizuki S., Prieto-Martin P., Nomura T., Sakaguchi N., Kohl J. (2010). Complement drives Th17 cell differentiation and triggers autoimmune arthritis. J. Exp. Med..

[47] LeibundGut-Landmann S., Gross O., Robinson M.J., Osorio F., Slack E.C., Tsoni S.V., Schweighoffer E., Tybulewicz V., Brown G.D., Ruland J. (2007). Syk- and CARD9-dependent coupling of innate immunity to the induction of T helper cells that produce interleukin 17. Nat. Immunol..

[48] Ruutu M., Thomas G., Steck R., Degli-Esposti M.A., Zinkernagel M.S., Alexander K., Velasco J., Strutton G., Tran A., Benham H. (2012). Beta-glucan triggers spondylarthritis and Crohn's disease-like ileitis in SKG mice. Arthritis Rheum..

[49] Hirota K., Yoshitomi H., Hashimoto M., Maeda S., Teradaira S., Sugimoto N., Yamaguchi T., Nomura T., Ito H., Nakamura T. (2007). Preferential recruitment of CCR6-expressing Th17 cells to inflamed joints via CCL20 in rheumatoid arthritis and its animal model. J. Exp. Med..

[50] Ito Y., Hashimoto M., Hirota K., Ohkura N., Morikawa H., Nishikawa H., Tanaka A., Furu M., Ito H., Fujii T. (2014). Detection of T cell responses to a ubiquitous cellular protein in autoimmune disease. Science.

[51] Korganow A.S., Ji H., Mangialaio S., Duchatelle V., Pelanda R., Martin T., Degott C., Kikutani H., Rajewsky K., Pasquali J.L. (1999). From systemic T cell self-reactivity to organ-specific autoimmune disease via immunoglobulins. Immunity.

[52] Matsumoto I., Staub A., Benoist C., Mathis D. (1999). Arthritis provoked by linked T and B cell recognition of a glycolytic enzyme. Science.

[53] Maccioni M., Zeder-Lutz G., Huang H., Ebel C., Gerber P., Hergueux J., Marchal P., Duchatelle V., Degott C., van Regenmortel M. (2002). Arthritogenic Monoclonal Antibodies from K/BxN Mice. J. Exp. Med..

[54] Ji H., Ohmura K., Mahmood U., Lee D.M., Hofhuis F.M.A., Boackle S.A., Takahashi K., Holerts V.M., Walport M., Gerard C. (2002). Arthritis critically dependent on innate immune system players. Immunity.

[55] Lee D.M., Friend D.S., Gurish M.F., Benoist C., Mathis D., Brenner M.B. (2002). Mast cells: A cellular link between autoantibodies and inflammatory arthritis. Science.

[56] Solomon S., Rajasekaran N., Jeisy-Walder E., Snapper S.B., Illges H. (2005). A crucial role for macrophages in the pathology of K/BxN serum-induced arthritis. Eur. J. Immunol..

[57] Auger J.L., Cowan H.M., Engelson B.J., Kashem S.W., Prinz I., Binstadt B.A. (2016). Brief Report: Arthritis in KRN T Cell Receptor-Transgenic Mice Does Not Require Interleukin-17 or Th17 Cells. Arthritis Rheumatol..

[58] Block K.E., Zheng Z., Dent A.L., Kee B.L., Huang H. (2016). Gut Microbiota Regulates K/BxN Autoimmune Arthritis through Follicular Helper T but Not Th17 Cells. J. Immunol..

[59] Katayama M., Ohmura K., Yukawa N., Terao C., Hashimoto M., Yoshifuji H., Kawabata D., Fujii T., Iwakura Y., Mimori T. (2013). Neutrophils are essential as a source of IL-17 in the effector phase of arthritis. PLoS ONE.

[60] Ivanov I.I., Atarashi K., Manel N., Brodie E.L., Shima T., Karaoz U., Wei D., Goldfarb K.C., Santee C.A., Lynch S.V. (2009). Induction of intestinal Th17 cells by segmented filamentous bacteria. Cell.

[61] Maeda Y., Takeda K. (2017). Role of Gut Microbiota in Rheumatoid Arthritis. J. Clin. Med..

[62] Iwakura Y. (2002). Roles of IL-1 in the development of rheumatoid arthritis: Consideration from mouse models. Cytokine Growth Factor Rev..

[63] Sutton C., Brereton C., Keogh B., Mills K.H., Lavelle E.C. (2006). A crucial role for interleukin (IL)-1 in the induction of IL-17-producing T cells that mediate autoimmune encephalomyelitis. J. Exp. Med..

[64] Ohtani T., Ishihara K., Atsumi T., Nishida K., Kaneko Y., Miyata T., Itoh S., Narimatsu M., Maeda H., Fukada T. (2000). Dissection of signaling cascades through gp130 in vivo: Reciprocal roles for STAT3- and SHP2-mediated signals in immune responses. Immunity.

[65] Sawa S., Kamimura D., Jin G.H., Morikawa H., Kamon H., Nishihara M., Ishihara K., Murakami M., Hirano T. (2006). Autoimmune arthritis associated with mutated interleukin (IL)-6 receptor gp130 is driven by STAT3/IL-7-dependent homeostatic proliferation of CD4+ T cells. J. Exp. Med..

[66] Li P., Schwarz M. (2003). The TNF-alpha transgenic mouse model of inflammatory arthritis. Springer Semin. Immunopathol..

[67] Aidinis V., Plows D., Haralambous S., Armaka M., Papadopoulos P., Kanaki M.Z., Koczan D., Thiesen H.J., Kollias G. (2003). Functional analysis of an arthritogenic synovial fibroblast. Arthritis Res. Ther..

[68] Lam J., Takeshita S., Barker J.E., Kanagawa O., Ross F.P., Teitelbaum S.L. (2000). TNF-α induces osteoclastogenesis by direct stimulation of macrophages exposed to permissive levels of RANK ligand. J. Clin. Investig..

[69] Zwerina K., Koenders M., Hueber A., Marijnissen R.J., Baum W., Heiland G.R., Zaiss M., McLnnes I., Joosten L., van den Berg W. (2012). Anti IL-17A therapy inhibits bone loss in TNF-α-mediated murine arthritis by modulation of the T-cell balance. Eur. J. Immunol..

[70] Miossec P. (2003). Interleukin-17 in rheumatoid arthritis: If T cells were to contribute to inflammation and destruction through synergy. Arthritis Rheum..

[71] Kolls J.K., Linden A. (2004). Interleukin-17 family members and inflammation. Immunity.

[72] Jovanovic D.V., Di Battista J.A., Martel-Pelletier J., Jolicoeur F.C., He Y., Zhang M., Mineau F., Pelletier J.P. (1998). IL-17 stimulates the production and expression of proinflammatory cytokines, IL-beta and TNF-α, by human macrophages. J. Immunol..

[73] Cho J.H., Gregersen P.K. (2011). Genomics and the multifactorial nature of human autoimmune disease. N. Engl. J. Med..

[74] Sakaguchi S. (2000). Regulatory T cells: Key controllers of immunologic self-tolerance. Cell.

[75] Bettelli E., Carrier Y., Gao W., Korn T., Strom T.B., Oukka M., Weiner H.L., Kuchroo V.K. (2006). Reciprocal developmental pathways for the generation of pathogenic effector TH17 and regulatory T cells. Nature.

[76] Pasare C., Medzhitov R. (2003). Toll pathway-dependent blockade of CD4+CD25+ T cell-mediated suppression by dendritic cells. Science.

[77] Valencia X., Stephens G., Goldbach-Mansky R., Wilson M., Shevach E.M., Lipsky P.E. (2006). TNF downmodulates the function of human CD4^+^CD25^hi^ T-regulatory cells. Blood.

[78] O’Sullivan B.J., Thomas H.E., Pai S., Santamaria P., Iwakura Y., Steptoe R.J., Kay T.W.H., Thomas R. (2006). IL-1 beta breaks tolerance through expansion of CD25+ effector T cells. J. Immunol..

[79] Izcue A., Hue S., Buonocore S., Arancibia-Carcamo C.V., Ahern P.P., Iwakura Y., Maloy K.J., Powrie F. (2008). Interleukin-23 restrains regulatory T cell activity to drive T cell-dependent colitis. Immunity.

[80] Komatsu N., Okamoto K., Sawa S., Nakashima T., Oh-hora M., Kodama T., Tanaka S., Bluestone J.A., Takayanagi H. (2014). Pathogenic conversion of Foxp3^+^ T cells into TH17 cells in autoimmune arthritis. Nat. Med..

[81] Wehrens E.J., Prakken B.J., van Wijk F. (2013). T cells out of control—Impaired immune regulation in the inflamed joint. Nat. Rev. Rheumatol..

[82] Noack M., Miossec P. (2014). Th17 and regulatory T cell balance in autoimmune and inflammatory diseases. Autoimmun. Rev..

[83] Lubberts E., Koenders M.I., van den Berg W.B. (2005). The role of T-cell interleukin-17 in conducting destructive arthritis: Lessons from animal models. Arthritis Res. Ther..

[84] Acosta-Rodriguez E.V., Napolitani G., Lanzavecchia A., Sallusto F. (2007). Interleukins 1beta and 6 but not transforming growth factor-beta are essential for the differentiation of interleukin 17-producing human T helper cells. Nat. Immunol..

[85] Cosmi L., De Palma R., Santarlasci V., Maggi L., Capone M., Frosali F., Rodolico G., Querci V., Abbate G., Angeli R. (2008). Human interleukin 17-producing cells originate from a CD161+CD4+ T cell precursor. J. Exp. Med..

[86] Ito Y., Usui T., Kobayashi S., Iguchi-Hashimoto M., Ito H., Yoshitomi H., Nakamura T., Shimizu M., Kawabata D., Yukawa N. (2009). Gamma/delta T cells are the predominant source of interleukin-17 in affected joints in collagen-induced arthritis, but not in rheumatoid arthritis. Arthritis Rheum..

[87] Noordenbos T., Yeremenko N., Gofita I., van de Sande M., Tak P.P., Canete J.D., Baeten D. (2012). Interleukin-17-positive mast cells contribute to synovial inflammation in spondylarthritis. Arthritis Rheum..

[88] Kirkham B.W., Lassere M.N., Edmonds J.P., Juhasz K.M., Bird P.A., Lee C.S., Shnier R., Portek I.J. (2006). Synovial membrane cytokine expression is predictive of joint damage progression in rheumatoid arthritis: A two-year prospective study (the DAMAGE study cohort). Arthritis Rheum..

[89] Yamada H., Nakashima Y., Okazaki K., Mawatari T., Fukushi J.I., Kaibara N., Hori A., Iwamoto Y., Yoshikai Y. (2008). Th1 but not Th17 cells predominate in the joints of patients with rheumatoid arthritis. Ann. Rheum. Dis..

[90] Smolen J.S., Agarwal S.K., Ilivanova E., Xu X.L., Miao Y., Zhuang Y., Nnane I., Radziszewski W., Greenspan A., Beutler A. (2017). A randomised phase II study evaluating the efficacy and safety of subcutaneously administered ustekinumab and guselkumab in patients with active rheumatoid arthritis despite treatment with methotrexate. Ann. Rheum. Dis..

[91] Adamopoulos I.E., Suzuki E., Chao C.C., Gorman D., Adda S., Maverakis E., Zarbalis K., Geissler R., Asio A., Blumenschein W.M. (2015). IL-17A gene transfer induces bone loss and epidermal hyperplasia associated with psoriatic arthritis. Ann. Rheum. Dis..

[92] Alzabin S., Abraham S.M., Taher T.E., Palfreeman A., Hull D., McNamee K., Jawad A., Pathan E., Kinderlerer A., Taylor P.C. (2012). Incomplete response of inflammatory arthritis to TNFalpha blockade is associated with the Th17 pathway. Ann. Rheum. Dis..

[93] Jones S.A., Sutton C.E., Cua D., Mills K.H. (2012). Therapeutic potential of targeting IL-17. Nat. Immunol..

[94] Kotake S., Yago T., Kobshigawa T., Nanke Y. (2017). The Plasticity of Th17 Cells in the Pathogenesis of Rheumatoid Arthritis. J. Clin. Med..

[95] Lyakh L., Trinchieri G., Provezza L., Carra G., Gerosa F. (2008). Regulation of interleukin-12/interleukin-23 production and the T-helper 17 response in humans. Immunol. Rev..

